# A Dynamic Mechanical Analysis on the Compatibilization Effect of Two Different Polymer Waste-Based Compatibilizers in the Fifty/Fifty Polypropylene/Polyamide 6 Blend

**DOI:** 10.3390/polym16172523

**Published:** 2024-09-05

**Authors:** Emilia P. Collar, Jesús-María García-Martínez

**Affiliations:** Polymer Engineering Group (GIP), Polymer Science and Technology Institute (ICTP), Spanish National Research Council (CSIC), C/Juan de la Cierva, 3, 28006 Madrid, Spain

**Keywords:** PA6/iPP, DMA, polymer blends, interfacial agents, polymers wastes, recycling, grafted polypropylenes, reactive processing, morphology stabilization

## Abstract

This study aims to examine the 50/50 polypropylene/polyamide 6 (iPP/PA6) system molded under confined flow conditions, both in its original state and after being modified by two different interfacial agents. This study provides two main insights. Firstly, it focuses on a polymer blend close to phase inversion. Secondly, it investigates the impact of using two different types of interfacial agents (derived from polymer waste) to enhance the compatibility between iPP and PA6. Dynamic Mechanical Analysis (DMA) has been employed to achieve these objectives. It is important to note that the investigation of the 50/50 iPP/PA6 system is a crucial focus predicted in previous studies, where a series of mechanical properties were evaluated using Box–Wilson design of experiments (DOEs) over the whole compositional range on the iPP/PA6 binary system. Thus, two interfacial modifiers, namely succinic anhydride (SA)-grafted atactic polypropylene with terminal, side, and bridge SA grafts (aPP-SASA) and succinyl-fluoresceine (SF) with bridge succinic anhydride grafting atactic polypropylene (aPP-SFSA), were employed. The authors obtained and characterized these agents. The quantity of these agents used in the blend was identified as a critical coordinate in prior studies conducted by the authors. The processing method used, compression molding under confined conditions, was chosen to minimize any orientation effect on the emerging morphology. All characterization procedures were performed on samples processed by contour machining to retain the blend morphologies as they emerged from the processing stage. Results from WAXS and SAXS synchrotron tests concluded there were no changes in the crystal morphology of the iPP or the PA6 in the blends nor any co-crystallization process throughout the compositional range. These findings, and the long period fits on the PP crystalline phase for the fifty/fifty blends we are discussing, will support the present DMA study. Finally, the efficiency of these interfacial modifiers has been concluded, even in this unfavorable scenario.

## 1. Introduction

Polymer-based materials have played a vital role in the quality of human life due to their extraordinary and functional properties, low cost, excellent durability, and so on [1]. This set of properties and potential applications has caused the global production of polymers to increase from 1.5 million tons in 1950 to nearly 400 million tons in 2021 [1]. Additionally, since more than fifty years ago, eco-balance, which jointly concerns both competitiveness in costs and environmental and sustainability requirements, addresses a large part of the R&D strategies used to look for new advanced organic-based materials by the enhancement of melt blending and compounding technologies of the currently available industrial thermoplastic polymers [2,3,4]. Indeed, understanding how thermal and flow fields induce changes in the structure of the blend components during processing, giving rise to each emerging morphology once it is in a solid state, remains a challenge. Additionally, the abovementioned environmental and sustainability requirements demand the most efficient ways to recover and recycle plastic waste, particularly from significant solid waste sources at the end-use of these materials. However, plastic materials almost always appear just compiled, giving rise to polymer mixtures with harmful properties after processing because of the complex and heterogeneous morphologies emerging from blends whose counterparts are immiscible [5,6], as is the case in our study.

Whatever the aim, the main challenge to understanding the emerging morphology of any heterogeneous organic polymer-based materials comes from the morphology-to-rheology solid coupling that characterizes the molten state of polymers [5]. Moreover, this mostly gives rise to the non-stability of the unmodified pristine blends, which, if co-continuous, tend to break up into a droplet/matrix type morphology, and if the latter, the trend towards recombination and coalescence on further processing operations gives rise to harmful or undesired properties. Therefore, compatibilizers or suitable interfacial modifiers are recommended to improve the blend quality and avoid the undesired recombination and coalescence effects on the results of further post-processing operations. Otherwise, morphology model predictions are highly complex as they approach the actual processing conditions and have limited practical applications, primarily when the wall mold significantly influences the emerging morphologies [6,7,8,9,10]. Hence, and as said before, getting complete control over the emerging morphologies from processing operations to assure their post-processing stability remains an open challenge [11]. In such a context, one finds that polyolefin-based materials, especially those based on polypropylene, stay at the edge of the commodity and the engineering polymer fields because of their enormous versatility and cost competitiveness [12,13]. Furthermore, this explains its presence as the main component in the plastic material fractions of the significant solid waste sources from big cities and automotive or agricultural uses, which become polypropylene- and ethylene-based polymers, with noticeable effects on plastic materials recycling policies [14,15,16]. Furthermore, major strategic application fields dealing with health, energy, or transport [17,18,19,20,21,22,23,24,25,26] account for the significant presence of the so-called nanostructured materials based on polypropylene [27,28].

All the abovementioned considerations hold a hot topic in studying the flow-induced emergent morphologies in those heterogeneous materials. Equally, the role played by the interfacial modifiers in their generation and stabilization, primarily when related to chemical reactions at the nanoscale domains of the interfaces [29,30,31]. Such studies involve the spatial region of interactions between the bordering chain segments at the different polymer domains, from a few atoms to several surface moieties, and actives at the interfacial ply depending on the stress/strain level undergone by the material along the processing history. It defines the interfacial ply by a finite thickness and a transport area at the nanoscale domain, which determines the material’s macroscopic behavior [32,33,34]. Hence, any interfacial phenomenon is restricted to the interface’s finite dimensions, implying an optimum compositional ratio to any suitable interfacial modifier that can optimize the material performance. Thus, and for economic reasons, it is all-advised to exceed such an optimum because the material properties would remain constant, if not worse, as mainly occurs [35]. Moreover, the abovementioned colossal complexity of the polymer flow explains the current absence of any practical application of the bottom-up theoretical models to predict the processing and emerging morphology of arbitrary polymer blends, let alone even a single polymer [11,36]. Thus, many years ago, we adopted a Top-Down approach, strongly supported by the employ of the Box–Wilson experimental design methodology, able to identify those optimal compositional ratios to obtain different polypropylene-based materials with deformable or non-deformable dispersed phases, respectively, blends or composites [32]. Therefore, the present work continues a previous morphological study by PPC TOM and FE SEM of a pristine 50/50 iPP/PA6 blend molded under confined flow conditions and modified by different amounts of either aPP-SASA- or aPP-SFSA-grafted polypropylenes [36]. That work discussed the different observed morphologies in light of the mechanical and dynamic thermal behaviors. Meanwhile, FT-IR spectroscopy concludes a chemical reaction between any end amine groups of PA6 and SA groups of aPP-SASA or aPP-SFSA [36]. We now discuss the dynamic mechanical (DMA) results of the fresh fifty/fifty iPP/PA6 blend and the ones modified by each of the two abovementioned different grafted atactic polypropylenes, obtained at the Polymer Engineering Group’s labs [37,38,39,40,41]. From an applied perspective, it is noteworthy that both the interfacial agents considered, based on grafted atactic polypropylene, match the requirements of the surfactant substances, with long sequences of non-polar or aliphatic species, chemically anchored to polar groups [41,42,43,44,45,46,47,48,49], which in turn also apply to the development of nanocomposites [50,51,52,53,54]. From such a perspective, the polyamide/polyolefin binary system is a case study of significant interest from many years ago because of its many industrial applications, mostly related to the decrease in the water absorption of polyamides by blending with polyolefins and the improvements in the blends’ impact resistance [52]. The fifty/fifty iPP/PA6 blend was identified as the critical compositional ratio close to the inversion phase point [36,54,55,56,57,58,59]. Other authors have also discussed this in the literature [55,60,61,62,63,64,65,66,67]. At this point, it is worth mentioning that most studies about compatibilization study PA6 contents lower than 50%. Some authors, based on random experiments, established that the 50/50 iPP/PA system did not suffer any changes by the presence of a maleic anhydride atactic polypropylene as a compatibilizer, concluding a null effect in the system [68]. However, the use of the Box–Wilson experimental design has demonstrated that this is not true by identifying this concentration as the critical coordinate and the capability of the interfacial agent to modify the 50/50 iPP/PA6 properties [43,56,57]. This evidences the risk of employing merely random experiments when studying complex systems to identify the actual performance of such a complex system. As mentioned before, these two immiscible phases (iPP and PA6) require enhancing the interactions at an interfacial level through a compatibilizer resembling one of the phases, especially for the more mobile (iPP) [32,56,57], looking for easy hosting of the interfacial agent. Additionally, it must be assumed that functionalities are excluded from the crystalline domains [37,54,55]. Consequently, the grafted groups have easier access to the amide groups in the PA6 phase and react with the succinic groups in the compatibilizer [37,54,55,56,57,58]. It is also noteworthy to mention that the interfacial agents used here (aPP-SASA and aPP-SFSA) have an amorphous origin [56,57,58], being then hosted into the amorphous phase of the semi-crystalline iPP, resulting in their interaction with the PA6 domains [43,55,56,57,58,59]. Moreover, we mention that both phases (iPP; PA6) are not rigid (but mobile), which also favors the interactions between the amide groups present in the polyamide and succinic groups in the interfacial agents [36,43,54,55,56,57,58]

The present discussion is focused on the relaxation spectra of the 50/50 iPP/PA6 blend and the variations associated with the interfacial changes induced by the different modifiers, and based on previous studies also identifying the interfacial agent critical concentration [36,43,54,55,56,57,58,59] supported by the Box–Wilson surface response method [63]. Additionally, previous WAXS and SAXS synchrotron results let us conclude the absence of either modification of the crystal morphology of iPP or PA6 in the blends or any co-crystallization process [32,64,65]. Finally, the long-period values for the fifty/fifty blends also support the present DMA discussion.

## 2. Materials and Methods

### 2.1. Materials

**Figure 1:**
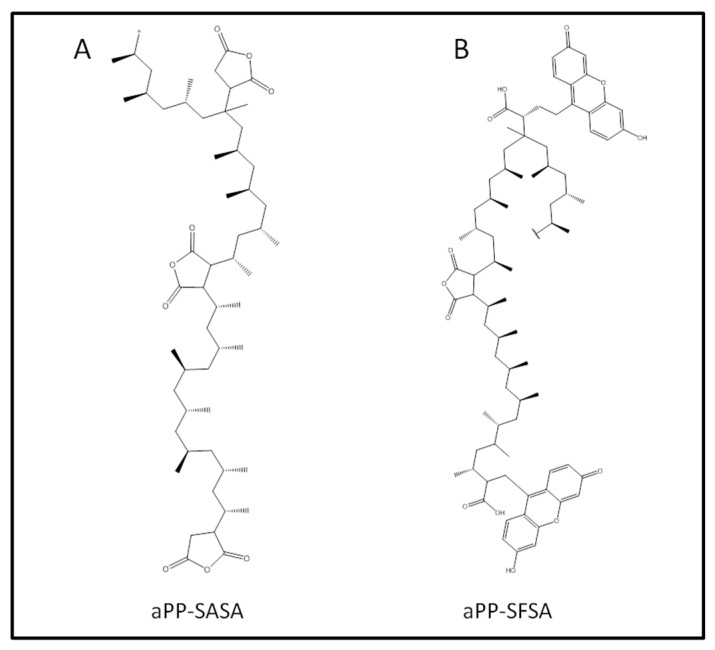
The chemical structure of the compatibilizers used in this work.

The homopolymers used in the present work were two commercial grades: Ultramid B3, Nylon 6, supplied by BASF (Spain), and Isplen 050, polypropylene, from Repsol Química (Spain). The declared properties for iPP as received were ρ = 0.90 g/cm^3^; Mw = 334,400 g/mol; Mn = 59,500 g/mol; Tm = 164 °C; and Tg = −13 °C. For the PA6, properties were ρ = 1.13 g/cm^3^; Mw = 25,000 g/mol; Mn = 13,000 g/mol; Tm = 221 °C; and Tg = 65 °C. The grafted polypropylenes were obtained at the authors’ laboratories by chemical modification of atactic polypropylene, a by-product of industrial propylene polymerization reactors, and supplied by Repsol Química (Spain). These compatibilizers were a succinic anhydride (aPPSASA) containing 3.05% (wt/wt) of grafted groups and a succinyl-fluoresceine succinic anhydride-grafted atactic polypropylenes (aPP-SFSA) with 6.2% of attached groups (expressed as succinic anhydride units). The detailed synthesis and characterization procedure were described to an extent elsewhere [37,38,39]. Figure 1 shows the chemical structures of both a-PP-SASA and a-PP-SFSA.

### 2.2. Sample Preparation

The compounds studied were a 50/50 iPP/PA6 fresh blend (without an interfacial agent) and a series of other interphases modified by the presence of 0.5, 9.0, or 17.5 *w*/*w* percent of either a-PP-SASA or a-PP-SFSA, respectively. Previously to the compounding, the PA6 pellets were conditioned for 48 h at 60 °C to remove any absorbed water. After that, the components (iPP, PA6, aPP-SASA, and aPP-SFSA) were dry mixed. After that, the blends were compounded in a Rheomix 600 chamber connected to a Rheocord 90 (Haake) at a temperature of 240 °C and 45 rpm mixing rate for 5 min once the torque was constant. Next, the blend obtained was cooled suddenly by immersion into ice water. Then, the material obtained was pelletized and then kept in a vacuum oven for at least 12 h. Afterward, 3 g of material was compression molded under confined flow conditions using a Dr. Collin press for 4 min at 270 °C, ensuring complete melting, and then for one additional minute at 10 MPa pressure and 270 °C. Then, the mold was passed to a cooling cartridge, which was applied with a pressure of 20 MPa (packaging step) until it reached room temperature. In this way, we obtained circular sheets (100 µm thick and 20 cm diameter). To avoid the preferential flow lines of the molding step, specimens were cut off in the circumferential sense of the sheets. The prismatic champions for DMA tests were 20 × 4 × 0.1 mm shaped. Before performing the DMA test, all the samples were conditioned for 12 h at room temperature and 50% relative humidity (RH). Two samples for each compound were tested by following the ASTM D5026 standards.

### 2.3. Characterization Procedures

All the experimental analyses were carried out over samples 100 ± 0.3 µm thick, machined from compression-molded sheets. They were virtually identical for all the testing procedures, with neither further manipulation nor processing other than the geometry and test dimensions machining. The tensile properties included here are merely informative, since they have been previously discussed in depth elsewhere [36,61].

Wide-angle and small-angle X-ray diffraction (WAXS and SAXS) were performed simultaneously using synchrotron radiation at the polymer beam line at Hasylab (DESY, Hamburg). The SAXS and WAXS measurements were obtained from the 100 µm samples described above at room temperature. The synchrotron beam was monochromatized by Bragg reflection through a single germanium crystal, resulting in a wavelength of 0.154 nm, focusing the beam in the horizontal direction. The vertical direction was concentrated with a Ni-mirror. The SAXS and WAXS signals were detected with two linear Gabriel detectors. The SAXS detector was placed at a distance of 235 cm from the sample and calibrated with the different orders of the long spacing of rat-tail cornea (L = 65 nm). The WAXS detector was calibrated by comparison with the crystalline diffraction peaks of PET (polyethylene terephthalate). More details concerning the instrument and the data analysis are given elsewhere [66,67]. The spectra were recorded with a diffraction angle (2θ) range of 10° to 32°.

**Figure 2:**
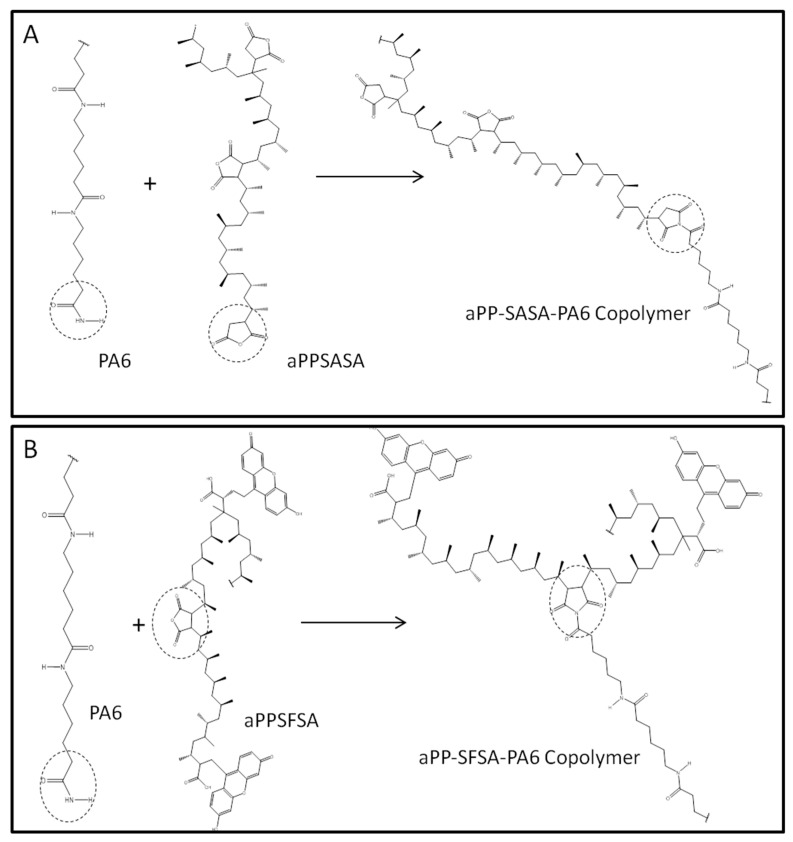
The proposed reaction between PA6 and aPP-SASA (**A**) or aPP-SFSA (**B**).

Molecular simulations on the two model copolymers’ behavior resulting from the reactive compatibilization between the amide groups in PA6 and the succinic units of the interfacial agents were performed by Chem3D^®^ (Perkin-Elmer) and using the MM2 interaction tool for minimizing the copolymer steric energy of the copolymers in Figure 2.

Dynamic mechanical (DMA) characterization was performed in a METTLER DMA861 analyzer under the tension mode and operating at a dynamic force of 0.2 N and a fixed frequency of 1 Hz to correctly identify interfacial effects [58,59]. To maintain the sample in the visco-elastic behavior range, a 4 μm amplitude and 2 °C/min heating rate were used. The temperature range varied between −60 °C and 140 °C. The low frequency and applied displacement avoid any morphological change provoked by eventual internal heat generation at other frequencies and displacements to avoid nonlinear responses [69].

## 3. Results and Discussion

### 3.1. Background

A previous study [36] thoroughly discussed the thermal and mechanical behavior of 50/50 iPP/PA6 pristine and modified blends under confined flow conditions. The study included an analysis of the morphology using TOM and FE SEM techniques. The TOM study focused on the bulk morphology of the blends after processing, while FE SEM analyzed the tensile fracture surfaces of the samples. The dynamic thermal and mechanical behavior during tensile testing at room temperature aligned with expectations from the reactive mixing process and compression molding under confined flow conditions influenced by the mold wall. This morphology study identified two primary factors affecting the DMA study of the different 50/50 iPP/PA6 blends. Firstly, the pristine and modified blends showed that iPP bore the unidirectional tensile load, attempting to reorient its amorphous regions by dragging the PA6 domains through a shear stress field across the interface. This resulted in highly strained iPP filaments parallel to the external load and unstrained areas with smooth surface voids left by the pullout of the PA6 domains. Secondly, in all the modified blends, there was a sharp decrease in the coalescence power of the PA6 domains during the molding step compared to the pristine blend. This decrease was attributed to reduced interfacial tension and emerging chemical bonds across the interface between the grafted SA groups and some end-amine groups of the PA6. This led to sensitive changes in the morphology of the PA6 domains, transitioning from a strained cylindrical or worm-like topography to ribbon-like topographies or spheroids/ellipsoids, depending on the specific blend. The 9% aPP-SASA 50/50 iPP/PA6-modified blend exhibited an optimal morphology characterized by the minimum particle size of the PA6 domains and the best mechanical properties [36]. Additionally, to support further discussion of the DMA spectra, some comments about the structure of the interfacial agents and the emerging copolymers, the morphological aspects, crystalline content, and mechanical properties are well worth reviewing [36,58,59,60,62,63].

#### 3.1.1. Molecular Modeling

**Figure 3:**
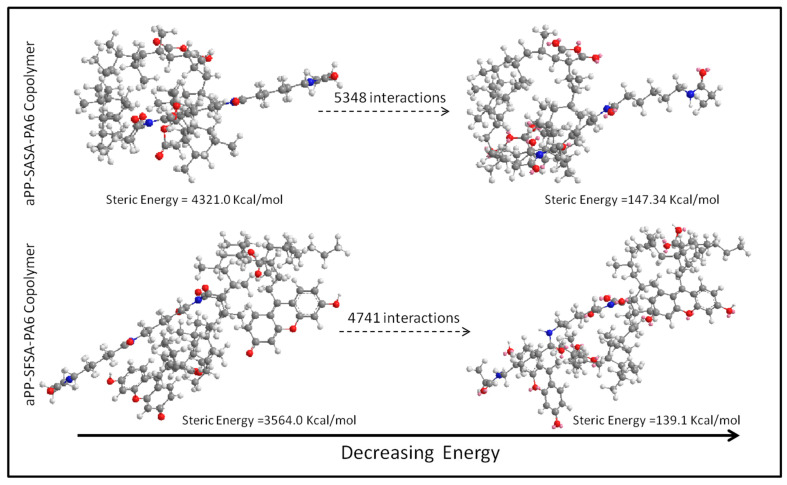
The molecular model with minimized steric energies for the indicated copolymers.

We performed preliminary modeling of the copolymers to obtain a rough idea of the different roles played by each interfacial agent (Figure 3).

We observe that in the case of the aPP-SASA-PA6 two arms copolymer (considering only a limited number of monomer units), the initial steric energy is around 4321 kcal/mol. By forcing it to have the more stable conformation, this passes to just 147.34 kcal/mol after 5348 interactions by using the minimizing energy tool (MM2) of the Chem3D^®^ software. Thus, we can observe a decrease in steric energy (ΔSE) of 4173.7 kcal/mol. Equally, the aPP-SFSA-PA6 three-arms copolymer passes from 3564 kcal/mol to 139.1 kcal/mol after 4741 interactions to reach the more stable conformation with a decrease in energy (ΔSE) of 3424.97 kcal/mol. We are keeping in mind that high steric energy means lower motion capability. The latter seems to corroborate a more significant number of chemical linkages between the amide groups of PA6 and the succinic units of the aPP-SASA, suggesting the higher efficiency of this compatibilizer in respecting aPP-SFSA in the iPP/PA 6 system. As previously mentioned, we conducted initial modeling of both the aPP-SASA-PA6 and the aPP-SFSA-PA6 copolymers to gain a rough understanding of their different potential configurations and spatial volume requirements. Using Chem3D^®^ software, we constructed the copolymers mentioned above by attaching the most available SA-grafted groups in each of the two interfacial modifiers to an end amine group located in a PA6 sequence, aiming to support the three types of grafted SA moieties (end, at the backbone, and both sides grafted at the aPP backbone) with the appropriate number of aPP monomer units. Based on the ball and rod models shown in Figure 3, it is evident that both models are presented alongside their respective steric energy values. The steric energy of the aPP-SASA two-armed copolymer is approximately 17.5% higher than that of the aPP-SFSA three-armed copolymer. Upon minimization, the aPP-SASA requires 8.8% more iterations than the aPP-SFSA, resulting in an end steric energy 5.5% higher than the latter. An exciting aspect of their respective localization at the PP/PA6 interphase is their different spatial conformations. The aPP-SASA exhibits an almost spherical conformation, whereas the aPP-SFSA does not show much conformational change. The SF moieties in the three-armed copolymer appear to envelop the neighborhood of the PA6 chain segment by diluting the aPP chain segments. In contrast, the former prevents such shielding by adopting an almost spherical arrangement of the aPP chain segments, regardless of whether they embody grafts.

Considering that a sphere represents the lowest surface value for a given volume, these observations are indeed consistent with our previous findings, particularly those related to the superior efficiency demonstrated by the aPP-SASA as an interfacial modifier at the PP/PA6 interphase and the saturation effect observed for the aPP-SFSA in the same role [36,58,59].

#### 3.1.2. X-ray Diffraction Studies

**Figure 4:**
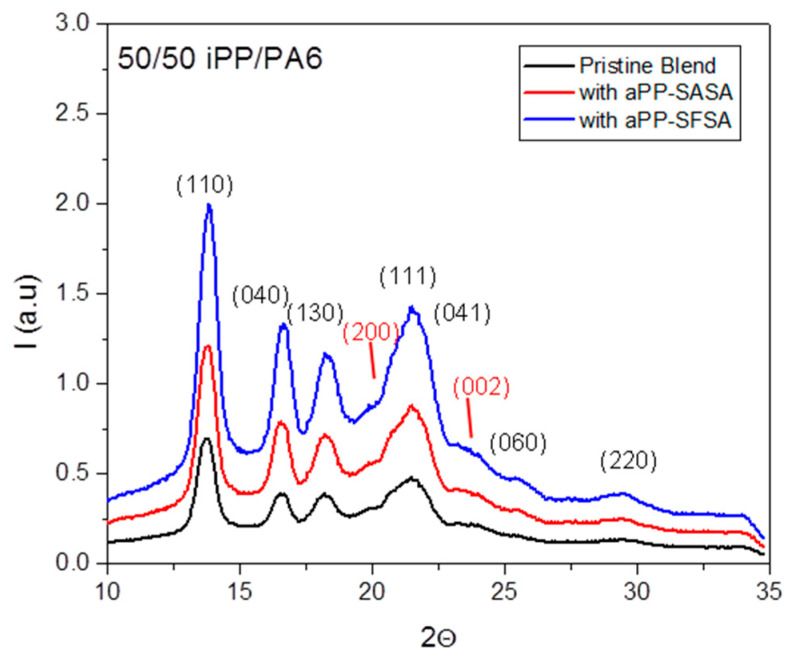
WAXS pattern for the indicated samples and with the notation of the α monoclinic planes for iPP (labeled in black) and PA6 (marked in red).

A series of WAXS and SAXS synchrotron experiments ascertain the proper experimental plan for processing history without any iPP and PA6 co-crystallization phenomena [32,69,70]. The WAXS patterns confirmed the non-co-crystallization of both polymers, but just the nucleation effect played for PA on the iPP phase [32,54,55,70,71]. Consequently, the iPP α monoclinic cells were well-characterized by the distinct peaks centered at the 2θ reflection angles of 14.2, 17.0, 18.8, 21.2, 22.0, 25.5, and 29.3^0^, corresponding to the (110), (040), (130), (111), (041), (060), and (220) α form crystallographic planes, respectively. Further, two weak peaks centered at 20 and 23^0^, typical of the (200) and (002) planes corresponding to the α form of PA6 [70,71,72,73,74,75,76], are identified in Figure 4.

**Figure 5:**
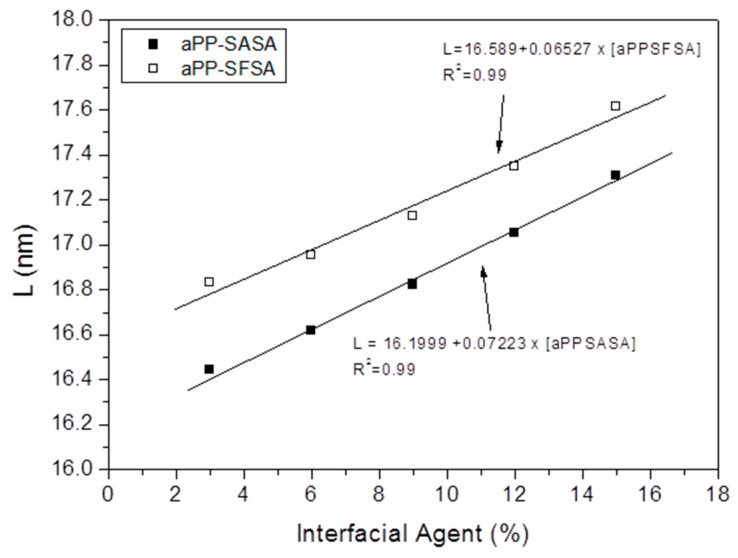
Evolution of the long spacing (L) with the interfacial content for the iPP in the 50/50 iPP/PA blends modified with aPP-SASA and aPP-SFSA.

These considerations about both the iPP and the PA6 crystalline phases are essential because of the high temperature undergone by the iPP phase during both the softening and the melting processes. Indeed, high processing temperatures decrease the molten viscosity and, in the absence of degradation, favor a high level of chain disentanglement, a crucial parameter in the relaxation modes at the DMA rubbery plateau region once well past the glass transition region. This latter is the case of the iPP, and it affects not only the amorphous but also the PP crystal/amorphous interface [64,65,74], as we could confirm by the changes in the iPP long-period (L) values in the modified blend by fitting the SAXS results (Figure 5).

L varies because the higher the amount of interfacial agent, the higher L for whatever interfacial agent is considered. Further, aPP-SFSA causes L values to be superior to those of aPP-SASA. Nevertheless, in both cases, the increasing presence of the interfacial agent provokes much higher values than the neat iPP (L = 15.4 nm) [64,65]. It is interesting to notice that the evolution of L follows a linear pattern with the increasing level of interfacial agent present in the blend, corresponding to both interfacial agents almost parallel each other, which means a nearly constant shift of 0.4 nm between L values of the respective systems (aPP-SFSA vs aPP-SASA in the blend).

#### 3.1.3. Tensile Properties

**Figure 6:**
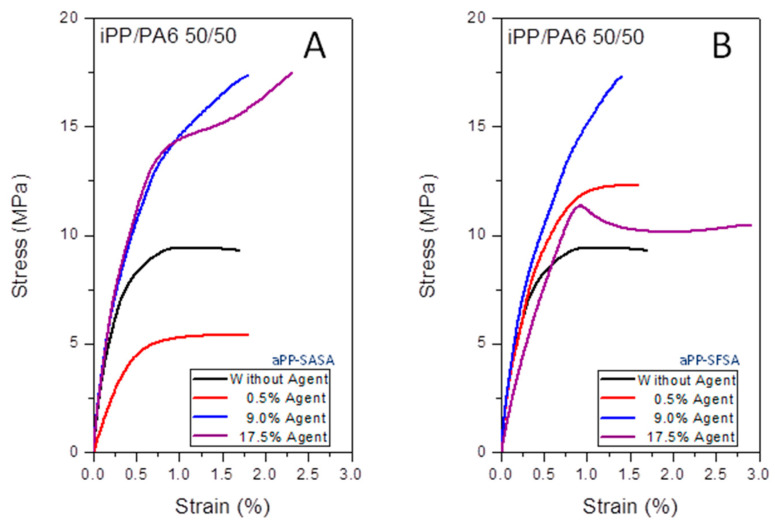
Tensile curves for the 50/50 iPP/PA6 blends with (**A**) aPP-SASA and (**B**) aPP-SFSA.

Figure 6 shows the tensile curves for the samples studied, wherein the very different patterns depend on the presence or absence of interfacial agents and the abruptly different effects on the mechanical properties of the 50/50 iPP/PA6 blends they cause. At a glance, we observe the very different behavior of both interfacial agents, confirming that these play an essential role even in the unfavorable scenario of the inversion phase. It is noteworthy to mention that except for the sample with 0.1% aPP-SASA, in all the cases, the mechanical parameters are improved, respecting the fresh blend, evidencing the efficient and differentiated role played by both interfacial agents.

In any case, in Table 1, we have compiled some mechanical parameters of the blend with and without an interfacial agent. More details about mechanical properties are published elsewhere [36,59].

It is perhaps noteworthy that they all appear in the same magnitude order as the fresh blend. This fact seems to evidence the debonding between the iPP and the PA6 domains, confirmed by the FE SEM observations in previous works. Thus, we can conclude the actual interfacial location of the interfacial agents in the modified blends [36].

### 3.2. Dynamic Mechanical Analysis

The background above will help the discussion of the studied samples from a DMA perspective.

#### 3.2.1. Unmodified iPP, PA6, and iPP/PA Blends

**Figure 7:**
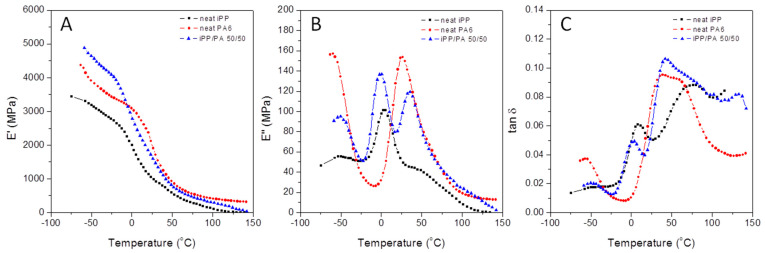
Evolution with the temperature of E′ (**A**), E″ (**B**), and the damp factor (**C**) for the pristine iPP, PA6, and the unmodified 50/50 iPP/PA6 blend.

The dynamic mechanical parameters of iPP, PA6, and the 50/50 iPP/PA6 blend are compiled in Figure 7. Plot A shows the thermal evolutions of the elastic component, E′, of the complex tensile modulus, E*. Plots B and C display the thermal evolutions of the corresponding loss component, E″, and the E″/E′ phase angle, respectively. As the temperature increases, the E′ curves for iPP and PA6 and their 50/50 blend decrease, following different slopes within different temperature ranges where the characteristic relaxations of both materials occur. The iPP E′ curve is lower than that of PA6, with three slope changes at −12.5, 18.2, and 63.7 °C. The PA6 E′ curve has up to six slope changes, three below 0 °C and three above it, similar to the 50/50 blend.

Notably, the blend’s slope changes are shifted upwards compared to those of PA6, except for those at −4.3 and 68.7 °C, which are the same. The blend’s curve is higher than the PA6 curve at low temperatures, crossing the PA6 E′ curve at the matching slope change at −4.3 °C. From that point on, the blend curve decreases almost parallel to the iPP curve until reaching the PA6 glass transition region, where all three E′ curves decrease almost parallel up to about 90 °C. At this highest temperature region, the iPP and the blend E′ curves decrease almost parallel, while the PA6 E′ curve decreases with a practically zero slope.

As anticipated, the changes in slope in the E′ curves correspond to the relaxation peaks observed in both the E″ curves (Figure 7B) and the tan δ curves (Figure 7C). At around −50 °C, the PA6 β relaxation peak was observed, attributed to local crankshaft-type motions involving an unbounded amide group near the chain ends and several methylene carbon groups [76,77,78,79,80]. The peak area of the PA6 β relaxation peak increases significantly with increasing water content in the PA6 phase. The tan δ plot of the fifty/fifty blend appears 5.5 °C higher than the single PA6 and shows a lower peak area than the homopolymer. Both effects are also observable in the corresponding E″ curves. This suggests a decrease in the population of “free” amide segments engaged at the PA6 domain borders. Moreover, the glass transition temperatures of the iPP and PA6 in the binary system appear 6.8 °C below and 4 °C above their respective individual transition temperatures [78,79,80].

As said above, each glass transition appears well defined on the respective E″ curve, which is noteworthy in the single iPP, given the almost symmetrical glass transition peak. Meanwhile, the right-side arm of the glass transition peak of the single PA6 shows a shoulder above 50 °C that breaks the peak’s symmetry. These considerations over both the E″ curves of the single homopolymers explain the modulation effect observed in the E″ plot of the pristine 50/50 blend characterized by a double peak curve in the overlapped response range of each one of the homopolymers involving the respective amorphous regions placed at the crystalline zones. Hence, in the single tan δ plot for iPP, we find a bi-modal curve with a first low temperature or glass transition peak placed at 8.8 °C, evolving as a symmetrical max–min up to 27 °C, increasing from then on as the temperature increases up to reach a broad maximum at 78 °C. This temperature region between 40 and 80 °C comprises the third level of relaxations in iPP [67]. In this so-called rubber–elastic transition, the former short-range diffusion motions at the chain-segments level responsible for the glass transition become available to those rapid short-range diffusion motions sharply depending on the molecular mass and the chain entanglement density. This relaxation zone may appear as a plateau or almost constant E′ values with increasing temperature or be identified by a significant slope smoothing. The single iPP E″ curve, Figure 7B plot, evolves in this region by following a short range of almost constant E″ values between 25 and 50 °C, decreasing again herein after by a continuous slope up to 110 °C, and decreasing then by a newly smoothed slope. The sharp increase of tan δ values at this temperature region agrees with the low entanglement density, previously pointed out because of the high processing temperature undergone by the single iPP. Concerning the PA6 phase at this temperature zone, the tan δ curve shows a broad glass transition peak in agreement with its low structural methylene to amide ratio [69,70,71], with a high-temperature shoulder at 59.6 °C, assigned to relaxation processes on large chain segments related to the PA6 crystalline phase that set free as the hydrogen bonding disappears. This agrees with a high crystalline content in the PA6 phase, which, according to the previously mentioned WAXS results, corresponds to its α form characterized by both the two reflections, the α_1_ at 20° related to the distance between hydrogen-bonded chains and the α_2_ at 23° to that one between hydrogen-bonded sheets [72]. By observing both the E″ and the tan δ plots of the iPP/PA6 50/50 blend, well-defined three peak curves appear, corresponding to the previously identified for the single iPP and PA6 main transitions. Hence, one may determine the respective “free” amorphous phases responsible for the iPP and the PA6 glass transitions over the tan δ curve in the Figure 7C plot. Indeed, the iPP glass transition peak in the blend appears to be 7 °C downwards shifted concerning the pristine iPP, in agreement with the decrease in the iPP crystalline content in the 50/50 blend [36,62]. Furthermore, it shows a lower peak area and then a lower amount of the iPP “free” amorphous phase involved in such transition. However, the glass transition of the PA6 in the 50/50 blend shows an upward shift of 4 °C, respecting the neat PA6. Meanwhile, the PA6 β transition region offers a similar and symmetrical inverted min–max evolution but a half-shortened concern than the single PA6. Furthermore, this peak appears 6 °C upwards shifted to that in the single PA6 and shows a sharp decrease in the corresponding peak area, to noteworthy for the almost coincident inflection point of both the max–min evolution of the β transition peak between the single PA6 and once in the 50/50 blend tan δ curves placed at 35 °C. Then, such a PA6 β transition shift well assigned to the “free” PA6 amorphous phase indicates a higher energy level to yield it and a decrease in the responsible PA6 population in the fifty/fifty blend. Otherwise, this was the cross point between the single iPP and the single PA6 tan δ curves, which seems to act as some inner reference in the blend tan δ curve. Indeed, one finds that the max–min peak looks almost symmetrical, with its lowest temperature arm overlapping with the tan δ curve of the single PA6 along 10 °C up to reach the minimum and passing then to nearly overlap with that of the single iPP while yielding the above-discussed glass transition. After falling to a minimum of 15 °C, the blend tan δ values increase again, entering the PA6 glass transition region, crossing the single tan δ PA6 curve at 33.8 °C, and reaching a maximum at 42.7 °C, four degrees above the glass transition temperature of the single PA6. Starting now, the curves go into the temperature range that enables the iPP and PA6 relaxations associated with their respective crystalline phases, appearing then the 50/50 blend tan δ curve as a wrapping curve of such relaxations. Therefore, over this curve, one may identify the PA6 shoulder previously exhibited by the neat PA6 curve, placed now at 67.3 °C matching with the cross point between both the PP and the PA6 tan δ curves and ten degrees overlapping, from 84 to 94 °C, with the decreasing arm of the broad peak of the single PP curve. Afterward, the 50/50 blend tan δ curve shows a subtle min–max evolution with two temperature peaks at 102.1 and 132.4 °C. These may be assigned to the dynamic mechanical response at the so-called β transition region of the iPP crystalline phase across the dynamic amorphous/crystal interface and would be strongly affected by the highly constrained amorphous phase present in the iPP, just placed between the PA6 domains borders and the grown around them iPP transcrystalline regions [73,74,75,76,77]. In summary, the precedent DMA discussion identifies the main relaxations of the iPP and the PA6 in the blend by referring each one to the corresponding neat polymers similarly processed while showing how the inner balance of their respective amorphous phases directly affects the overall dynamic mechanical response. Such a balance accounts for at least two different kinds of amorphous regions: on the one side, the “free” amorphous phase placed at the bulk of the respective iPP and PA6 domains and able to yield each one of their respective glass transitions [78,79,80]. On the other hand, those amorphous regions at the borders of the iPP and the PA6 domains are highly constrained for the former because of the nucleation effect of the PA6 domains and the subsequent transcrystalline regions growing during the iPP crystallization when processed under dynamic conditions. Under the next section, we try to correlate the DMA responses of the modified 50/50 iPP/PA6 blends with the interfacial role played by either the aPP-SASA or the aPP-SFSA, both able to change the morphology and then the properties of the pristine binary system [36,62].

#### 3.2.2. DMA of the Modified 50/50 iPP/PA6 Blends

**Figure 8:**
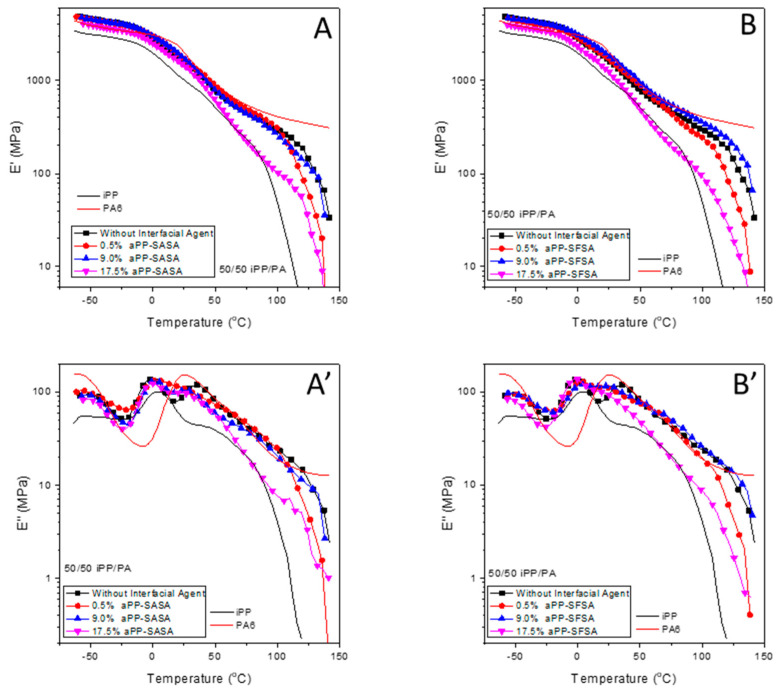
Evolution of E′ (**A**,**A’**), and E″ (**B**,**B’**) with temperature for the indicated samples.

Figure 8 compiles the semi-log E′ and E″ plots for the aPP-SASA, left hand side, and the aPP-SFSA, right hand side, respectively modified iPP/PA6 blends. The semi-log scales modulate the lowest temperature region for E′ and E″ values while magnifying those above room temperature. The semi-log E′ plots of the modified blend, likely to the pristine one, evolve almost parallel to that of the single PP by approaching, first, the single PA6 E′ values and further to the single iPP ones with increasing amount of the interfacial modifier. The effect is higher in the aPP-SFSA-modified blends than in the aPP-SASA-modified ones. It continues up to the surroundings of −4.3 °C, formerly found as some inner reference in the binary system, which identified the cross point between the pristine blend and the single PA6 E′, which passed to yield the highest E′ values after this. Hence, at the temperature region between 35 °C and 55 °C, we point out the differences between the aPP-SASA-modified blends whose E′ values almost match those of the single PA6 E′ at the lowest modifier amount (0.51%). In contrast, the aPP-SFSA-modified ones match these later, at 0.51% and 9% of the modifier. Otherwise, the highest amount of both of the two modifiers (17.5%) does not match with the single PA6 E′ values, but sharply approaches the single iPP E′ values, matching them between 65 and 72 °C the aPP-SASA-modified blend, and between 40 °C and 47 °C the aPP-SFSA-modified one.

Moreover, both yield lower E′ values than the neat iPP, at 14 °C and 41 °C, respectively (86 °C for the former, and up to 88 °C for the latter). Finally, for the temperature region above 100 °C, the PA6 E′ values decrease with an almost zero slope up to the test end. In contrast, the E′ values of any of the modified blends decrease following significant and nearly parallel slopes. The result is very close to that of the pristine iPP, being the 9%-modified blends that yield the highest E′ values at the highest test temperature region and even surpassing those of the pristine blend, as is the case for the 9% aPP-SFSA-modified blend. Of course, these results find their correspondence in the semi-log E″ plots, which show their most significant changes at the main relaxation zones of each single homopolymer as the pristine blend did. Hence, concerning the effect of the amount of each one of the interfacial modifiers, it is worth noting the almost overlapping area between the E″ curves of the 0.5% and the 9.0% aPP-SFSA-modified blends, and likewise the splitting of all the aPP-SA/SA-modified ones, all along the temperature axis.

**Figure 9:**
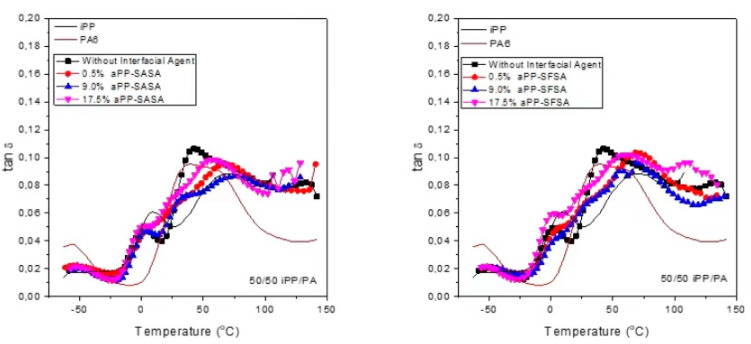
Evolution of the damp factor with temperature for the indicated samples.

In addition, the overall smoothness trend observed in the signal curves of the modified blends is also of interest concerning those of the neat homopolymers and those of the pristine blend. The variations are especially noticeable at the respective iPP and PA6 glass transition peaks of each modified blend, which show significantly decreased peak areas, indicating a lower population of “free” amorphous phase in the modified blends than in the pristine one. This agrees with the expected increase in those amorphous regions engaged at the iPP/PA6 interface, especially those just involved in the transcrystalline iPP regions. Furthermore, Figure 9, which compiles the peak temperatures identified all along the tan δ curves for the modified blends, lets us compare the different effects of each one of the modifiers on the primary relaxations of the pristine blend. Hence, concerning the PP glass transition, all the aPP-SASA-modified blends show the exact same peak temperature, 2.3 °C, just 0.3 °C above that of the pristine blend; meanwhile, the aPP-SFSA-modified ones show an upward shift of 6.5 °C for the 0.5 and the 9% modified blends, and only of 1 °C the 17.5%-modified one. Conversely, the PA6 glass transition peak in all the modified blends appears to have shifted downwards, concerning that in the pristine blend. All the aPP-SFSA-modified blends appear 10.3 °C below. Meanwhile, the aPP-SASA-modified ones appear, successively, at 1.7, 2.6, and 9.2 °C with increasing amounts of aPPSASA.

## 4. Conclusions

This work lets us conclude the efficiency of two interfacial agents obtained from polypropylene polymerization wastes: aPP-SASA and aPP-SFSA. Conversely to that which is said in the very few articles in the literature undertaken on the study of the iPP/PA6 system with high PA6 contents, this work undoubtedly demonstrates the very noticeable effect of the interfacial agents on the final properties of the 50/50 iPP/PA6 system (close to the phase inversion). Additionally, this effect differs depending on the type of grafted groups in the interfacial agent. This study has considered this unusual proportion based on previous works by the authors that identified the 50/50 blend as the critical coordinate by employing a quadratic Box–Wilson statistical design of experiments implying complexity. In contrast to what is usually written in the literature, the very adverse scenario that the inversion phase presents is sensitive to the effect of the interfacial modifiers. This fact informs us about the risk of using random experiments to conclude the complex behavior of polymer-based systems. This effect has been established here mainly by dynamic mechanical analysis and mechanical and X-ray observations. These results agree with previous studies by the authors concerning thermal, vibrational spectroscopy, tensile properties, X-rays, and morphology analysis. These DMA results also agree with the preliminary molecular simulation models performed here related to forming block aPP-SASA-PA6, or the three-armed aPP-SFSA-PA6 copolymers at the iPP/PA6 interface.

## Figures and Tables

**Table 1:** Mechanical parameters for the indicated blends.

Interfacial Agent	iPP/Interfacial Agent (*w*/*w* %)	Strength at Yield (MPa)	Strain at Yield (%)	Strength at Break (MPa)	Strain at Break (%)
None	50/0 (fresh iPP/PA6)	9.4 ± 0.1	0.70 ± 0.16	9.3 ± 0.1	1.51 ± 0.10
aPP-SASA aPP-SFSA	50/0.1	5.4 ± 0.1 12.3 ± 0.2	0.70 ± 0.12 1.31 ± 0.10	5.4 ± 0.1 12.3 ± 0.3	1.82 ± 0.18 1.61 ± 0.20
aPP-SASA aPP-SFSA	50/9.0	15.0 ± 0.1 17.0 ± 0.1	0.91± 0.12 1.33 ± 0.16	17.2 ± 0.1 17.3 ± 0.1	1.93 ± 0.21 1.42 ± 0.18
aPP-SASA aPP-SFSA	50/17.5	14.5 ± 0.2 11.9 ± 0.1	0.81 ± 0.08 0.92 ± 0.09	17.5 ± 0.2 10.5 ± 0.1	2.31 ± 0.21 2.92 ± 0.23

## Data Availability

Other Data than those in the article also available.

## References

[1] Pernot H., Baumert M., Court F., Leibler L. (2002). Design and properties of co-continuous nanostructured polymers by reactive blending. Nat. Mater..

[2] Kornfield J.A., Kumaraswamy G., Issaian A.M. (2002). Recent advances in understanding flow effects on polymer crystallization. Ind. Eng. Chem. Res..

[3] Utracki L.A. (1989). Polymer Alloys and Blends.

[4] Tadmor C.G., Gogos C.G. (2006). Principles of Polymer Processing.

[5] Cristini V., Hooper R.W., Macosko C.W., Simeone M., Guido S. (2002). A numerical and experimental investigation of lamellar blend morphologies. Ind. Eng. Chem. Res..

[6] Van Puyvelde P., Vananroye A., Cardinaels R., Moldenaers P. (2009). Review on morphology development of immiscible blends in confined shear flow. Polymer.

[7] Camesasca M., Manas-Zloczower I. (2009). Danckwerts revisited-The use of entropy to define scale and intensity of segregation. Macromol. Theory Simul..

[8] Berezkin A.V., Guseva D.V., Kudrayavtsev V. (2012). Formation of linear and graft copolymers at a Polymer/polymer interface: How copolymer brush and microdomain morphology control heterogeneous reactions. Macromolecules.

[9] de Gennes P.G. (1980). Conformation of polymers attached to an interface. Macromolecules.

[10] Baschnagel J., Meyer H., Varnik F., Metzger S., Aichele M., Müller M., Binder K. (2003). Computer simulations of Polymers close to solid interfaces: Some selected topics. Interf. Sci..

[11] Tonzani S. (2013). The renaissance of Polyolefins. J. Appl. Polym. Sci..

[12] Galli P., Vecellio G. (2004). Polyolefins: The Most Promising Large-Volume Materials for the 21st Century. J. Polym. Sci. Polym. Chem..

[13] Gardiner F. (2010). Plastics and Environment.

[14] Collar E.P., García-Martínez J.M., Olabisi O., Adewale K. (2016). Environment, Health and Safety: Regulatory and Legislative Issues. Handbook of Thermoplastics.

[15] García-Martínez J.M., Collar E.P., Olabisi O., Adewale K. (2016). Recycling of Thermoplastics. Handbook of Thermoplastics.

[16] Anjum N., Gulrez SK H., Singh H., Gupta B. (2006). Development of antimicrobial polypropylene sutures by graft polymerization. I. Influence of grafting conditions and characterization. J. Appl. Polym. Sci..

[17] Toth R., Ferrone M., Miertus S., Chiellini E., Fermeglia M., Priel S. (2006). Structure and energetics of biocompatible polymer nanocomposite systems: A molecular dynamics study. Biomacromolecules.

[18] Poon B.C., Chum S.P., Hiltner A., Baer E. (2004). Modifying adhesion of linear low-density polyethylene to polypropylene by blending with a homogeneous ethylene copolymer. J. Appl. Polym. Sci..

[19] Poon B.C., Chum S.P., Hiltner A., Baer E. (2004). Adhesion to polyethylene blends to polypropylene. Polymer.

[20] Hiltner A., Liu RY F., Hu Y.S., Baer E. (2005). Oxygen transport as a solid state structure probe for polymeric materials: A review. J. Polym. Sci. Part B Polym. Phys..

[21] Jarus D., Hiltner A., Baer E. (2002). Barrier properties of polypropylene/polyamide blends produced by microlayer extrusion. Polymer.

[22] Jang B.N., Costache M., Wilkie C.A. (2005). The relationship between thermal degradation behavior of polymer and the fire retardancy of polymer/nanoclay nanocomposites. Polymer.

[23] Song P., Shen Y., Du B., Peng M., Shen L., Fang Z. (2009). Effects of Reactive Compatibilization on the morphological, thermal, mechanical, and rheological properties of intumescent flame retardant polypropylene. Appl. Mater. Interfaces.

[24] Lomakin S.M., Zaikov G.E., Koverzanova E.V. (2005). Thermal degradation and combustibility of polypropylene filled with magnesium hydroxide micro-filler and polypropylene nano-filled aluminosilicate composite. Oxid. Commun..

[25] Abetz V., Brinkmann T., Dijkstra M., Ebert K., Fritsch D., Ohlrogge K., Paul D., Peinemann K.-V., Pereira-Nunes S., Scharnagl N. (2006). Developments in membrane research: From material via process design to industrial application. Adv. Eng. Mater..

[26] Presting H., König U. (2003). Future nanotechnology developments for automotive applications. Mater. Sci. Eng..

[27] Gahleitner M. (2011). Polyolefins for the 21st century. Express Polym. Lett..

[28] Joanny J.F. (2003). Polymer at Interfaces. Interface Sci..

[29] Huang J., Ichinose I., Kunitake T. (2005). Nanocoating of natural cellulose fibers with conjugated polymers: Hierarchical polypyrrole composite materials. Chem. Commun..

[30] Chong K.P. (2004). Nanoscience and engineering. J. Phys. Chem. Solids.

[31] García-Martínez J.M., Areso S., Taranco J., Collar E.P., Kyu T., Nwabunma D. (2008). Heterogeneous Materials Based on Polypropylene. Polyolefin Blends.

[32] Collar E.P., García-Martínez J.M. (2010). On Chemical Modified Polyolefins by Grafting of Polar Monomers. A Survey Based on Recent Patents Literature. Recent Pat. Mater. Sci..

[33] Ruiz-Pérez L., Royston J., Patrick J., Fairclough A., Ryan A.J. (2008). Toughening by nanostructure. Polymer.

[34] Sinha Ray S., Okamoto M. (2003). Polymer/layered silicate nanocomposites: A review from Preparation to processing. Prog. Polym. Sci..

[35] Allegra G., Raos G., Vacatello M. (2008). Theories and simulations of polymer-based nanocomposites: From chain statistics to reinforcement. Prog. Polym. Sci..

[36] Collar E.P., Areso S., Taranco J., García-Martínez J.M. (2015). Understanding the Morphological Changes in the Polypropylene/Polyamide 6 Fifty/Fifty Blends by InterfacialModifiers Based on Grafted AtacticPolypropylenes: Microscopic, Mechanical, and Thermal Characterization. J. Polym..

[37] García Martínez J.M., Areso S., Collar E.P. (2006). The transient nature of maximum maleic anhydride grafting on polypropylene: A mechanistic approach based on a consecutive reaction model. I. Batch solution process. J. Appl. Polym. Sci..

[38] García Martínez J.M., Areso S., Collar E.P. (2007). The transient nature of maximum maleic anhydride grafting on polypropylene: A mechanistic approach based on a consecutive reaction model. II. A comparison between the batch solution and molten state process. J. Appl. Polym. Sci..

[39] García Martínez J.M., Areso S., Laguna O., Collar E.P. (1999). FT-IR Quantitative characterization of chemically modified polypropylenes containing succinic grafted groups. J. Appl. Polym. Sci..

[40] García Martínez J.M., Areso S., Laguna O., Collar E.P. (1999). Changes on glass transition temperature in atactic polypropylene containing polar groups. J. Therm. Anal. Calorim..

[41] O’Shaughnessy B., Sawhney U. (1996). Reaction kinetics at polymer-polymer interfaces. Macromolecules.

[42] O’Shaughnessy B., Vavylonis D. (2003). Irreversibility and polymer adsorption. Phys. Rev. Lett..

[43] Sadiku-Agboola O., Sadiku E.R., Adegbola A.T., Biotidara O.F. (2011). Rheological properties of polymers: Structure and morphology of molten polymer blends. Mater. Sci. Appl..

[44] Cardinaels R., Vananroye A., Puyvelde P.V., Moldenaers P. (2011). Breakup criteria for confined droplets: Effects of compatibilization and component viscoelasticity. Macromol. Mater. Eng..

[45] Ajji A., Utracki L.A. (1996). Interphase and compatibilization of Polymer Blends. Polym. Eng. Sci..

[46] Van Puyvelde P., Oommen Z., Koets P., Groeninckx G., Moldenaers P. (2003). Effect of reactive compatibilization on the interfacial slip in nylon-6/EPR blends. Polym. Eng. Sci..

[47] Shi D., Ke Z., Yang J., Gao Y., Wu J., Yin J. (2002). Rheology and morphology of reactively compatibilized PP/PA6 blends. Macromolecules.

[48] Duvall J., Sellitti C., Myers C., Hiltner A., Baer E. (1994). Effect of compatibilization on the properties of PP/PA66 (75/25 wt/wt) blends. J. Appl. Polym. Sci..

[49] Cheng M.H., Balazs A.C., Yeung C., Ginzburg V.V. (2003). Modeling reactive compatibilization of a binary blend with interacting particles. J. Chem. Phys..

[50] Chow W.S., Abu Bakar A., Mohd Ishak A.A., Karger-Kocsis J., Ishiaku U.S. (2005). Effect of maleic anhydride-grafted ethylene-propylene rubber on the mechanical, rheological and morphological properties of organoclay reinforced polyamide 6/polypropylene nanocomposites. Eur. Polym. J..

[51] Ciardelli F., Coiai S., Passaglia E., Pucci A., Ruggeri G. (2008). Nanocomposites based on polyolefins and functional thermoplastic materials. Polym. Int..

[52] Zenga Q.H., Yua A.B., Lub G.Q. (2008). Multiscale modeling and simulation of polymer nanocomposites. Prog. Polym. Sci..

[53] Gahleitner M. (2001). Melt rheology of Polyolefins. Prog. Polym. Sci..

[54] Ginzburg V.V., Peng G., Qiu F., Jasnow D., Balazs A.C. (1999). Kinetic model of phase separation in binary mixtures with hard mobile impurities. Phys. Rev. E.

[55] Hietaoja P.T., Holsti-Miettinen R.M., Seppälä J.V., Ikkala O.T. (1994). The effect of viscosity ratio on the phase inversion of polyamide 66/polypropylene blends. J. Appl. Polym. Sci..

[56] Gabriele M., Pasquino R., Grizzuti N. (2011). Effects of viscosity-controlled interfacial mobility on the coalescence of immiscible polymer blends. Macromol. Mater. Eng..

[57] González-Montiel A., Keskkula H., Paul D.R. (1995). Morphology of nylon 6/ polypropylene blends compatibilized with maleated polypropylene, Journal of Polymer Science. Polym. Phys..

[58] García-Martínez J.M., Collar E.P. (2020). The Role of a Succinyl Fluorescein-Succinic Anhydride Grafted Atactic Polypropylene on the Dynamic Mechanical Properties of Polypropylene/Polyamide-6 Blends at the Polypropylene Glass Transition. Polymers.

[59] García-Martínez J.M., Collar E.P. (2019). Industrial Waste Origin Succinic anhydride grafted atactic polypropylene as compatibilizer of full range Polypropylene/Polyamide 6 Blends as Revealed by Dynamic Mechanical Analysis at the Polypropylene Glass Transition. Polym. Eng. Sci..

[60] Verdier C., Vinagre HT M., Piau M., Joseph D.D. (2000). High temperature interfacial tension measurements of PA6/PP interfaces compatibilized with copolymers using a spinning drop tensiometer. Polymer.

[61] García Martínez J.M., Areso S., Collar E.P. (2001). Structure Development on PA6/PP Blends. Interfacial modifications and preliminary modellization starting on macroscopic properties. J. Macromol. Sci. Part B Phys..

[62] García Martínez J.M., Marco C., Areso S., Collar E.P. (2001). Thermal behavior of PA6/PP blends under dynamic conditions. The effect of different interfacial agents based on chemically modified atactic polypropylene. J. Macromol. Science. Part B Phys..

[63] Box G.E.P., Hunter W.G., Hunter J.S. (1978). Statistics for Experimenters.

[64] García Martínez J.M., Areso S., Gómez M.A., Marco C., Collar E.P. Role of Succinic Anhydride Grafted Atactic Polypropylene (a-PP-SA) on the Melting and Crystallization Behaviour under Dynamic Conditions of the PP Phase in Interfacial Modified Polypropylene/Polyamide 6 Blends. A Synchrotron WAXS and SAXS Study. HASYLAB Annual Report 2002 Part I. http://hasyweb.desy.de/science/annual_reports/2002_report/index.html.

[65] García Martínez J.M., Areso S., Gómez M.A., Marco C., Collar E.P. On the Changes of the PP Phase in Interfacial Modified PP/PA6 by Means of Succinic Anhydride or Succinyl-Fluoresceine/Succinic Anhydride Grafted Atactic Polypropylenes. A Synchrotron WAXS and SAXS Comparative Study. HASYLAB Annual Report 2003 Part I. http://hasyweb.desy.de/science/annual_reports/2003_report/index.html.

[66] Zhao Z., Yu W., Liu Y., Zhang J., Shao Z. (2004). Isothermal crystallization behaviors of nylon 6/montmorillonite nanocomposite. Mater. Lett..

[67] Phillips P.J. (1990). Polymer crystals. Rep. Prog. Phys..

[68] Wu M.-H., Wang C.-C., Chen C.-Y. (2020). Chemical modification of atactic polypropylene and its applications as a crystallinity additive and compatibility agent. Polymer.

[69] McCrum N.G., Read B.E., Williams G. (1967). Anelastic and Dielectric Effects in Polymer Solids.

[70] Ong E.S., Kim Y., Williams L. (1986). Dynamic Mechanical Properties of some nylon and their Blends. J. Appl. Polym. Sci..

[71] Liang Z., Williams H.L. (1992). Dynamic Mechanical properties of Polypropylene-Polyamide Blends: Effect of Compatibilization. J. Appl. Polym. Sci..

[72] Kohan M. (1995). Nylon Plastics Handbook.

[73] García-Martínez J.M., Areso S., Taranco J., Collar E.P. (2009). Dynamic Mechanical Analysis of the interfacial changes in Polypropylene/Talc composites induced by Different Interfacial Modifications from the Reinforcement side. J. Appl. Polym. Sci..

[74] Pepin J., Miri V., Lefebvre J.M. (2016). New insights into the Brill Transition in Polyamide 11 and Polyamide 6. Macromolecules.

[75] Holmes D.R., Bunn C.W., Smith D.J. (1955). The Crystal Structure of Polycaproamide: Nylon 6. J. Polym. Sci..

[76] McCrum N.G., Buckley C.P., Bucknall C.B. (1997). Principles of Polymer Engineering.

[77] Passaglia E., Martin G.M.J. (1964). Dependence of mechanical relaxation on morphology in isotactic polypropylene. Res. Natl. Bur. Stand..

[78] Struick L.C.E. (1987). The mechanical and physical ageing of semicrystalline polymers: 1. Polymer.

[79] Struick L.C.E. (1987). The mechanical behaviour and physical ageing of semicrystalline polymers: 2. Polymer.

[80] Van Krevelen D.W. (1992). Properties of Polymers.

